# Inequalities in access to NHS primary care dental services in Scotland during the COVID-19 pandemic

**DOI:** 10.1038/s41415-023-5856-z

**Published:** 2023-05-24

**Authors:** Abodunrin Q. Aminu, Alex D. McMahon, Claire Clark, Andrea Sherriff, Caroline Buchanan, Chris Watling, Ahmed Mahmoud, Shauna Culshaw, William Mackay, Megan Gorman, Raymond Braid, Maura Edwards, David I. Conway

**Affiliations:** 225184130101660224150 School of Medicine, Dentistry, and Nursing, University of Glasgow, Glasgow, UK; Public Health Scotland, Edinburgh and Glasgow, United Kingdom; 587969130864224689224 Public Health Scotland, Edinburgh and Glasgow, United Kingdom; 879583970241927527150 Public Health Scotland, Edinburgh and Glasgow, UK; School of Health and Life Sciences, University of the West of Scotland, Paisley, United Kingdom

## Abstract

**Introduction** This study aimed to quantify the impact of the COVID-19 pandemic on access and inequalities in primary care dental services among children and adults in Scotland.

**Methods** Access was measured as any NHS Scotland primary care dental contacts derived from administrative data from January 2019 to May 2022, linked to the area-based Scottish Index of Multiple Deprivation for children and adults, and related to population denominator estimates from National Record Scotland. Inequalities for pre-pandemic (January 2019-January 2020) and recent (December 2021-February 2022, and March 2022-May 2022) periods for both children and adults were calculated and compared using the slope index of inequality and relative index of inequality.

**Results** Following the first lockdown (March 2020) there was a dramatic fall to near zero dental contacts, followed by a slow recovery to 64.8% of pre-pandemic levels by May 2022. There was initial widening of relative inequalities in dental contacts in early 2022, which, more recently, had begun to return to pre-pandemic levels.

**Conclusion** COVID-19 had a major impact on access to NHS primary dental care, and while inequalities in access are apparent as services recover from lockdown, these inequalities are not a new phenomenon.

**Supplementary Information:**

Zusatzmaterial online: Zu diesem Beitrag sind unter 10.1038/s41415-023-5856-z für autorisierte Leser zusätzliche Dateien abrufbar.

## Key points

- Access to NHS primary dental care in Scotland dramatically reduced due to COVID-19 public health measures.
- De-escalation of infection and prevention control measures for dental practices were only commenced from April 2022.
- By May 2022, NHS primary care services had not completely recovered to the pre-pandemic level of patient contacts.
- It is important to continue to monitor inequalities in access to dental services and to ensure that policies promote equity as services continue to recover from COVID-19.

**Supplementary Information:**

Zusatzmaterial online: Zu diesem Beitrag sind unter 10.1038/s41415-023-5856-z für autorisierte Leser zusätzliche Dateien abrufbar.

## Introduction

COVID-19 was declared a pandemic by the World Health Organisation on 11 March 2020,^1^ and a UK-wide first national lockdown was announced on 23 March 2020 to prevent the community spread of the SARS-CoV-2 virus.^2^ The first national lockdown in Scotland came to an end 28 May 2020 and was replaced with phased level restrictions dependent on the rate of infection in each of Scotland's regions.^3^ As the infection rate began to rise once more towards the end of 2020, restrictions were increased and tightened during the holiday season, eventually leading to the second lockdown in mainland Scotland on 5 January 2021.^3^ However, by 25 January 2021, the first vaccination programme against COVID-19 had started in Scotland and children were gradually allowed to return to schools and nurseries from 22 February 2021.^3^ Further restrictions were lifted on 2 April 2021 and there was no longer a requirement to 'stay at home'.^3^ On July 19 2021, most of the restrictions were withdrawn for all of Scotland, and by 9 August 2021, only a few protective measures remained (such as testing, use of face coverings, and two metre physical distancing in healthcare settings).^3^ On 3 December 2021, community transmission of the Omicron variant in Scotland was confirmed. Additional recommendations for decreasing social contacts were made starting on 14 December 2021 and continued through the winter and into the spring, and there was no requirement to wear face-coverings on public transportation and in most interior situations from 8 April 2022.

**Fig. 1:**
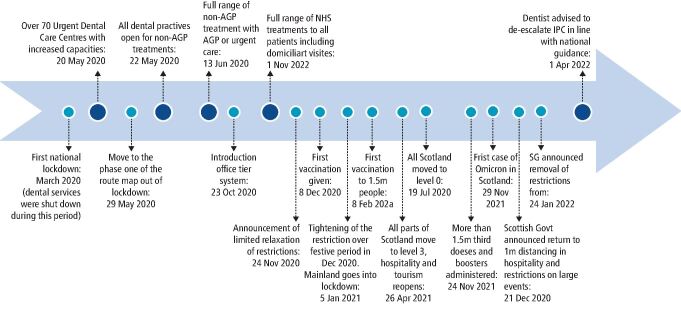
Timeline of COVID-19 in Scotland, including changes in IPC guidance for dental practices

As shown in Figure 1, the public health measures introduced during the pandemic also included components that directly related to healthcare settings.^2^^,^^3^ NHS Scotland was put on an 'emergency footing' on March 17 2020 during the first national lockdown and NHS dental practices were closed due to the anticipated risks of transmission associated with receiving dental care.^3^ In Scotland, the National Infection Prevention and Control Manual was updated several times during the pandemic to control the number of cases and reduce harm from the virus.^4^ Over 70 urgent dental care centres were established for the provision of emergency dental treatment and there was remobilisation of primary care NHS dental services starting from 20 May 2020 for patients with acute and essential oral health care needs.^5^ Dental practices were permitted to reopen for face-to-face consultation with patients requiring urgent dental care treatments that could be provided using non-aerosol generating procedures (AGPs) from 22 June 2020,^6^ and from 13 July 2020, dentists were able to see patients for the full range of routine non-AGP dental care.^7^ From 17 August 2020, aerosol-associated treatments were permitted for urgent dental care only.^7^ Practices were able to provide the full range of NHS treatments to all patients in need of both urgent and non-urgent care and dentists were also able to provide domiciliary care from 1 November 2020.^8^ Dentists were allowed to de-escalate their infection prevention and control measures in line with national guidance from the Chief Nursing Officer from 1 April 2022^9^ to alleviate system pressures and allow an increase in patients' dental access throughout.^9^^,^^10^

Prior to the pandemic, socioeconomic inequalities existed in oral health and dental access across different populations.^11^ In Scotland, while registration levels were high and the population could potentially access dental care (with minimal inequalities in registration), participation rates varied by level of socioeconomic deprivation, with those from the most socioeconomically deprived areas less likely to access/contact dental services regularly. For instance, in September 2019, the percentage of children (79.9%) and adult patients (62.1%) from the most socioeconomically deprived areas who participated (attended) was lower compared to the 89.0% children and 71.6% adult levels from the least deprived communities.^12^

Recent publications have shown that dental care access and oral health inequalities might present differently during the pandemic.^13^^,^^14^ Despite the impact of the COVID-19 pandemic on health services and health inequalities across the world and the UK, the full scale of the impact on oral health and oral health inequalities, including access to dental care, has yet to be fully understood. Here, we aimed to investigate the impact of the pandemic on access to NHS primary care dental services in the population overall and in relation to different socioeconomic groups across Scotland.

## Materials and methods

NHS primary care dental services data from the Management Information and Dental Accounting System database were requested from Public Health Scotland for the period January 2019 to February 2022, and subsequently updated to include data for the period March 2022 to May 2022.

To measure access to NHS primary care dental services, we used the number of claims per months (per episode of care) made by primary care dental teams to the NHS as our primary outcome in adults and children separately and together (henceforth, referred to as dental 'contacts'). These claims are made by both the general dental service and public dental service.^13^ We compared the median number of contacts for each period and divided these into three periods for the data analysis: the pre-pandemic period (January 2019 to January 2020), the first evaluation period (December 2021 to February 2022), and second evaluation period (March 2022 to May 2022), including the patients' area-based Scottish Index of Multiple Deprivation (SIMD) categories for children (<18 years) and adults (18+ years). Population denominators for the same period were obtained from National Records Scotland.

The SIMD 2020 is a relative measure of socioeconomic deprivation across over 6,900 data zones (small areas) in Scotland.^15^ This index uses data from seven domains: income; employment; education; health; access to services; crime; and housing, to rank each of the data zones. In the present study, SIMD quintiles were used to categorise the data zones into fifths, with SIMD 1 representing the 20% most socioeconomically deprived areas and SIMD 5 the 20% least deprived fifth.

The trends in NHS primary care dental contacts among children and adults over the COVID-19 pandemic period in Scotland were plotted using line graphs. Inequalities in the frequency of dental contacts with NHS primary dental care for children and adults in this study were assessed via calculating both the Slope Index of Inequality (SII) and Relative Index of Inequality (RII). Inequality measures were calculated based on SIMD and compared between the pre-pandemic period and the first evaluation period and also between the pre-pandemic period and the second (more recent) evaluation period.

The SII is a slope coefficient for the population that describes the association between health status or frequency of health conditions across socioeconomic scale or categories.^16^ It reflects the socioeconomic variable that has been rescaled to 0 (highest) and 1 (lowest) and can be interpreted as the absolute difference in health status or frequency of health conditions between the socioeconomic hierarchy. The SII responds to changes in the mean of the population or frequency of the health problems. It can be limited in predicting the inequality gap when comparing socioeconomic differences in different populations. SII was calculated in the present study as the absolute difference overall in the median monthly percentage of contacts with NHS primary care dental care between the most deprived communities to the least deprived communities (assuming linearity).

The RII is used to examine relative inequality gaps and can be calculated by dividing the SII by the population mean or frequency of the health condition.^16^ RII was calculated in the present study by dividing the SII by the weighted mean of the percentage of primary care dental contacts in the population for each timepoint.

To achieve the inequality analysis, the median percentages of dental contacts and SIMD were utilised for the pre-pandemic period (January 2019 to January 2020), first evaluation period (December 2021 to February 2022) and the second evaluation period (March 2022 to May 2022). The median monthly percentages were calculated and linked to the patients' home area-based SIMD level, categorised as SIMD 1 (most deprived fifth) and SIMD 5 (least deprived fifth) for children and adults separately. All analyses were conducted in SAS version 9.4 (SAS, Cary, NC).

## Results

**Fig. 2:**
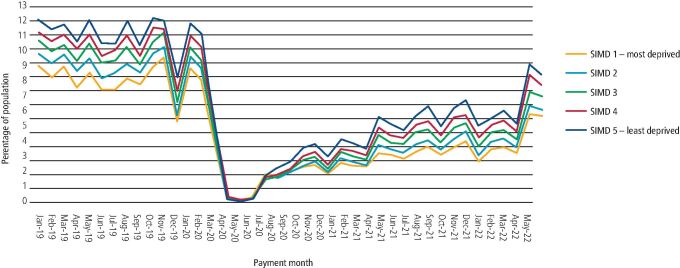
Percentage of the child population that had contact with primary care NHS dental services by payment month of claim and SIMD fifth, January 2019 to May 2022 (the percentage axis has been shortened to highlight the differences between SIMD fifths)

Contacts in the NHS primary care dental services dropped to near zero between the period of March 2020 and June 2020 during the first national lockdown for both children (Fig. 1) and adults (Fig. 2). Following easing of the COVID-19 public health measures, these contacts slowly began to increase. Table 1 shows the overall number of these contacts by month for children and adult populations for the pre-pandemic period (January 2019 to January 2020), the first evaluation period (December 2021 to February 2022), and the second evaluation period (March 2022 to May 2022). The median of monthly contacts in the first evaluation period was 49,432 claims for children and 177,632 claims for adults compared to the pre-pandemic median of 109,126 and 374,400 contacts, respectively. In the second evaluation period, the median of monthly contacts for children had risen further to 74,861, 68.6% of pre-pandemic levels, and for adults to 240,185, 64.1% of the pre-pandemic activity. The median of the combined monthly contacts for children and adults was 315,046 in the second evaluation period compared to the pre-pandemic 486,114 contacts, indicating a 64.8% service recovery overall.

**Table 1:** Number of dental contacts by payment month of claim - January 2019 to May 2022*

Period	Month, year	Children	Adults	Total
Pre-pandemic period	January 2019	112,583	411,017	523,600
	February 2019	105,889	346,443	452,332
	March 2019	111,714	374,400	486,114
	April 2019	99,090	333,966	433,056
	May 2019	111,251	399,693	510,944
	June 2019	96,529	345,896	442,425
	July 2019	98,087	338,043	436,130
	August 2019	109,126	376,883	486,009
	September 2019	98,090	349,292	447,382
	October 2019	113,696	383,534	497,230
	December 2019	71,397	262,533	333,930
	January 2020	110,900	414,948	525,848
Median		109,126	374,400	486,114
First evaluation period	December 2021	40,669	156,852	197,521
	January 2022	49,432	181,652	231,084
	February 2022	51,860	177,632	229,492
Median		49,432	177,632	229,492
Second evaluation period	March 2021**	45,073	140,599	185,672
	April 2022**	79,911	253,133	333,044
	May 2022	74,861	74,861	315,046
Median		74,861	240,185	315,046
Key: * = note that this table is the abridged version (Supplementary Table A shows the complete data); data was not available for Nov 2019 ** = note that March 2022 data was taken mid-month and some of the March data were reported with April 2022 data.				

At the population level, inequalities in access to NHS primary dental care were evident before the pandemic. In January 2019, 10.2% of the overall population in the least socioeconomically deprived areas (SIMD 5) had contact with primary care NHS dental services compared to 8.1% in the most socioeconomically deprived areas (SIMD 1) (see online Supplementary Tables B).

**Fig. 3:**
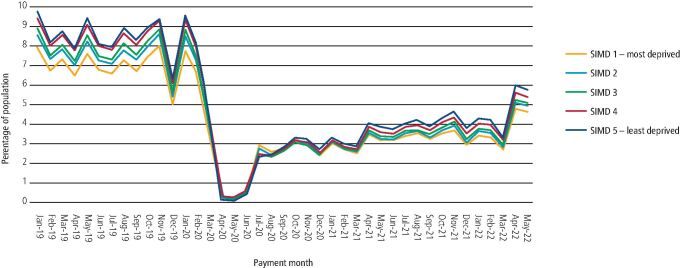
Percentage of the adult population that had contact with primary care NHS dental services by payment month of claim and SIMD fifth, January 2019 to May 2022 (the percentage axis has been shortened to highlight the differences between SIMD fifths)

Figure 2 and Figure 3 show the percentage of those that had contact with NHS primary care dental services by payment month and SIMD quintile between January 2019 and May 2022 for the children and adult population, respectively. Pre-pandemic, the monthly median percentage of the child population that had contacts was 8.3% for those in SIMD 1 and 11.9% for SIMD 5. For the adult population, monthly median percentage contacts pre-pandemic was 7.3% for those in SIMD 1 and 8.8% for SIMD 5. In the most recent three months (second evaluation period), the monthly median percentage of the child population that had contacts was 5.7% for those in SIMD 1 and 8.5% for SIMD 5, while in the adult population, contact levels were 4.7% for SIMD 1 and 5.8% for SIMD 5.

Table 2 shows that the pre-pandemic SII was 4.55% and 1.84% for children and adults, respectively, and by the first evaluation period (December 2021 to February 2022) had reduced to 2.67% and 1.07%, respectively. By the second, more recent evaluation period (March 2022 to May 2022), the SII had increased to 3.66% for children and 1.32% for adults. This indicates a reduction in absolute inequalities from pre-pandemic levels, which would be expected due to the overall reduced levels of activity across all SIMD fifths, with the trend in recent months for absolute inequalities beginning to return to their pre-pandemic levels.

**Table 2:** The pre-pandemic (Jan 2019 - Jan 2020), first evaluation period (Dec 2021 - Feb 2022) and second evaluation period (Mar 2022 -May 2022) median number of NHS primary care dental contacts for children and adults by SIMD, including SII and RII

Population	Pre-pandemic (Jan 2019 - Jan 2020)				Post (Dec 2021 - Feb 2022)					Post (March 2022 - May 2022)		
	SIMD	Contacts	Pop	%	Contacts		Pop		%	Contacts	Pop	%
Children	1	19,154	229,708	8.34	8,155		228,626		3.57	13,088	228,626	5.72
	2	18,855	203,474	9.27	8,283		202,700		4.09	12,618	202,700	6.22
	3	19,764	190,320	10.4	8,882		189,586		4.68	13,436	189,586	7.09
	4	22,802	204,068	11.14	10,689		205,362		5.2	16,121	205,362	7.85
	5	23,914	201,592	11.86	11,289		200,648		4.55	17,106	200,648	8.53
	Difference SIMD 5 - 1			3.52					0.98			2.81
	Ratio SIMD 5:1			1.42					1.27			1.49
	SII [95%CI]			4.55[3.85-5.25]					2.67[2.38-2.95]			3.66[3.06-4.25]
	RII [95%CI]			0.45[0.38-0.52]					0.58[0.52-0.64]			0.52[0.43-0.60]
Adults	1	62,825	856,405	7.34	28,867		854,101		3.38	39,863	854,101	4.67
	2	69,139	883,310	7.83	31,906		881,076		3.62	43,779	881,076	4.97
	3	73,254	901,557	8.13	33,742		902,095		3.74	45,950	902,095	5.09
	4	77,634	902,572	8.6	36,494		909,199		4.01	49,078	909,199	5.4
	5	78,387	890,294	8.8	38,024		892,607		4.26	51,620	892,607	5.78
	Difference SIMD 5 - 1			1.46					0.88			1.11
	Ratio SIMD 5:1			1.19					1.26			1.24
	SII [95%CI]			1.84[1.40-2.28]					1.07[0.85-1.29]			1.32[0.94-1.71]
	RII [95%CI]			0.23[0.17-0.28]					0.28[0.22-0.34]			0.26[0.18-0.33]

However, the RII rose for children from 0.45 pre-pandemic to 0.58 in the first evaluation period but started to fall thereafter (0.52 in the second evaluation period). For adults, RII was lower than children, but followed a similar trajectory: 0.23 pre-pandemic, rising to 0.28 at the first evaluation period, then 0.26 at the second evaluation period. The overlapping confidence intervals for adult RII indicate that there was no significant change in inequalities across the three periods.

## Discussion

This study covers dental contacts with NHS dental primary care services across Scotland between January 2019 and May 2022. The data show that the level of dental contacts fell to a record low shortly after March 2020 during the national lockdown and by May 2022 was still only 64.8% of the pre-pandemic level, with the child population having a slightly higher overall level of recovery (68.6%) compared with the level among the adult population (64.1%). Our findings also show that there was an initial slight widening of relative inequalities in early 2022, with those from the most affluent areas being more likely to receive the more limited available dental contacts. However, by May 2022, this was not quite back to pre-pandemic levels of inequality observed in the levels of dental contacts.^11^^,^^12^

The closure of dental services as part of the initial public health measures taken in response to curb the spread of COVID-19 infection was a widely utilised approach internationally.^17^ This inevitably resulted in dramatic falls in dental access in many countries. Our results, showing near zero dental access during the first national lockdown, was consistent with data published for England^14^ and reports from other countries, including Germany,^18^ France^19^ and Australia.^20^ Okike *et al.* reported that an estimated 365,000 infants in the UK from the previous year birth cohort missed their first dental visit because of the pandemic in 2020.^21^

There are several potential implications associated with reduced access on this scale. There were several reports of changes in reported oral health risk behaviours during the pandemic,^14^ including increases in sugar consumption from 8% to 15% in UK households,^22^ with the corresponding associated potential increases in oral diseases and need or demand for dental services coming at a time of dramatically reduced dental services. However, there is still not sufficient empirical data to assess the full impacts of the pandemic on oral health at the population level.^23^ Our study begins to contribute to this data and evidence gap providing detailed analysis of access to NHS primary care dental services in Scotland, which, before the pandemic, provided over 90% of dental services in Scotland.^12^

The assessment of inequalities using the area-based deprivation index has been reliably established in the literature.^24^^,^^25^ The pattern shown from our results suggest that there was an initial widening of relative inequalities in dental access, which have begun to return to the pre-pandemic levels. A USA study reported that the distribution of caries preventive services became less accessible to children from low-income families (39%) during the pandemic compared to those from the higher-income families (46%).^26^ The Childsmile national child oral health improvement programme for Scotland provided preventive services in multiple settings, including community, nursery, and school, as well as via NHS dental practices.^27^ These have all been substantially impacted by the pandemic^13^ and are likely to also have knock-on impacts on child oral health and further exacerbate persistent child oral health inequalities.

In addition to dental service supply side factors, there are multiple other factors likely associated with the reduction in dental care utilisation during the pandemic, which are also interlinked with the root causes of health inequalities.^28^ Aside from public health-related restrictions during the lockdown, the worsening socioeconomic situation for many individuals and other factors, such as perceived barriers and changes in health behaviours, might have also contributed to the reduction in dental access.^29^ The ability to access and travel to healthcare services during the pandemic was influenced by individuals' perception of need and risk.^29^ Even when there was a provision for urgent dental care, people were caught in between the decision to attend dental services or stay away to avoid getting infected with COVID-19.

Overall, our results describe the trends in access to primary care dental services in Scotland and associated inequalities during the pandemic. The strength of our study lies in the high quality, completeness and national coverage of NHS Scotland primary dental care services and its associated claims data,^12^ which were available for analysis on a monthly basis. However, this study is limited in not including other types of dental contacts that were increasingly utilised during the pandemic, such as the NHS 24 services, hospital dental services, urgent dental care centres, and tele-dentistry (internet or web application communications).^30^ Moreover, there are no available data on the levels of private dental services provided during or indeed before the pandemic, which are likely to vary for different socioeconomic groups. Analyses also did not include information on the nature of dental treatment provided within the contact.

## Conclusion

The COVID-19 pandemic had a huge impact on dental access, with NHS primary dental care services remaining lower than pre-pandemic levels before the time of de-escalation of infection prevention and control measures in dental settings. Although, inequalities in access have all but returned to pre-existing levels and these data shine a new light on persisting inequality in access to dental services.

It is important to continue to monitor inequalities in access to dental services and to ensure that policies promote equity as services continue to recover from the pandemic and in future dental service reform.

## Supplementary Information

Supplementary Information (PDF 276KB)
