# Supplementary material for: Inequalities in access to NHS primary care dental services in Scotland during the COVID-19 pandemic
Source: Br Dent J. 2023 May 24:1–6. Online ahead of print. doi: 10.1038/s41415-023-5856-z (PMC10208681; doi:10.1038/s41415-023-5856-z)
Supplement: Supplementary file 1 — Supplementary Information (PDF 276KB) [file 41415_2023_5856_MOESM1_ESM.pdf]

Supplementary Table A

Number of dental contacts by payment month of claim; January 2019 to May 2022*

		Number of claims			% of 2019 counts		
**Year**	**Month**	**Children**	**Adults**	**Total**	**Children**	**Adults**	**Total**
2019	January	112,583	411,017	523,600			
2019	February	105,889	346,443	452,332			
2019	March	111,714	374,400	486,114			
2019	April	99,090	333,966	433,056			
2019	May	111,251	399,693	510,944			
2019	June	96,529	345,896	442,425			
2019	July	98,087	338,043	436,130			
2019	August	109,126	376,883	486,009			
2019	September	98,090	349,292	447,382			
2019	October	113,696	383,534	497,230			
2019	November	#N/A	#N/A	#N/A			
2019	December	71,397	262,533	333,930			
2020	January	110,900	414,948	525,848	98.5	101.0	100.4
2020	February	101,103	343,811	444,914	95.5	99.2	98.4
2020	March	47,263	188,560	235,823	42.3	50.4	48.5
2020	April	3,619	10,569	14,188	3.7	3.2	3.3
2020	May	1,475	7,993	9,468	1.3	2.0	1.9
2020	June	3,365	23,299	26,664	3.5	6.7	6.0
2020	July	20,022	137,809	157,831	20.4	40.8	36.2
2020	August	20,965	125,354	146,319	19.2	33.3	30.1
2020	September	25,109	138,247	163,356	25.6	39.6	36.5
2020	October	31,833	164,721	196,554	28.0	42.9	39.5
2020	November	34,357	151,805	186,162	#N/A	#N/A	#N/A
2020	December	25,958	124,698	150,656	36.4	47.5	45.1
2021	January	37,408	158,628	196,036	33.2	38.6	37.4
2021	February	34,139	137,391	171,530	32.2	39.7	37.9
2021	March	31,534	128,639	160,173	28.2	34.4	32.9
2021	April	48,671	181,435	230,106	49.1	54.3	53.1
2021	May	44,406	165,642	210,048	39.9	41.4	41.1
2021	June	41,302	161,387	202,689	42.8	46.7	45.8
2021	July	49,093	176,424	225,517	50.1	52.2	51.7
2021	August	52,912	180,852	233,764	48.5	48.0	48.1
2021	September	43,422	167,281	210,703	44.3	47.9	47.1

2021	October	52,923	183,712	236,635	46.5	47.9	47.6
2021	November	57,911	197,000	254,911	#N/A	#N/A	#N/A
2021	December	40,669	156,852	197,521	57.0	59.7	59.2
2022	January	49,432	181,652	231,084	43.9	44.2	44.1
2022	February	51,860	177,632	229,492	49.0	51.3	50.7
2022	March	45,073	140,599	185,672	40.3	37.6	38.2
2022	April	79,911	253,133	333,044	80.6	75.8	76.9
2022	May	74,861	240,185	315,046	68.6	64.1	64.8

Supplementary Tables B

Combined children and adults dental contacts by SIMD quintile: Number of patients and % of population seen by GDS/PDS; by payment month and SIMD quintile; Scotland

		SIMD 1 - most deprived		SIMD 2		SIMD 3		SIMD 4		SIMD 5 - least deprived		SIMD Unknown
Year	Month	No. of patients	% of pop.	No. of patients	% of pop.	No. of patients	% of pop.	No. of patients	% of pop.	No. of patients	% of pop.	No. of patients
2019	January	88,493	8.1	95,734	8.8	100,932	9.2	108,221	9.8	111,226	10.2	799
2019	February	77,008	7.1	83,686	7.7	87,097	8.0	94,530	8.5	96,382	8.8	601
2019	March	84,096	7.7	89,808	8.3	93,234	8.5	100,436	9.1	102,301	9.4	773
2019	April	73,312	6.7	80,142	7.4	83,328	7.6	91,432	8.3	91,921	8.4	708
2019	May	85,889	7.9	93,102	8.6	98,349	9.0	105,908	9.6	109,754	10.1	737
2019	June	75,576	7.0	81,217	7.5	85,400	7.8	92,195	8.3	93,588	8.6	682
2019	July	74,015	6.8	80,418	7.4	84,195	7.7	91,248	8.2	92,227	8.4	678
2019	August	81,839	7.5	87,917	8.1	93,709	8.6	101,318	9.2	104,543	9.6	705
2019	September	75,816	7.0	82,336	7.6	85,940	7.9	92,980	8.4	95,294	8.7	722
2019	October	84,760	7.8	91,352	8.4	95,474	8.7	103,500	9.4	105,224	9.6	779
2019	November	91,673	8.4	97,906	9.0	101,196	9.3	107,813	9.7	108,196	9.9	859

2019	December	55,820	5.1	59,956	5.5	64,023	5.9	70,627	6.4	73,769	6.8	594
2020	January	88,311	8.2	96,510	8.9	101,255	9.3	109,645	9.8	111,167	10.2	798
2020	February	75,458	7.0	81,685	7.5	85,422	7.8	93,749	8.4	95,265	8.7	573
2020	March	41,648	3.8	43,771	4.0	46,194	4.2	49,316	4.4	49,677	4.5	380
2020	April	3,011	0.3	2,872	0.3	2,944	0.3	3,014	0.3	1,866	0.2	39
2020	May	2,292	0.2	2,001	0.2	1,653	0.2	1,795	0.2	1,290	0.1	13
2020	June	5,647	0.5	5,055	0.5	4,557	0.4	4,587	0.4	4,492	0.4	19
2020	July	29,952	2.8	27,953	2.6	25,828	2.4	26,383	2.4	24,611	2.3	181
2020	August	26,449	2.4	25,346	2.3	24,554	2.2	25,831	2.3	26,402	2.4	215
2020	September	28,055	2.6	27,707	2.6	27,950	2.6	29,785	2.7	30,661	2.8	252
2020	October	33,303	3.1	32,929	3.0	33,492	3.1	35,547	3.2	37,203	3.4	244
2020	November	31,003	2.9	31,314	2.9	32,482	3.0	35,370	3.2	37,110	3.4	201
2020	December	25,803	2.4	26,062	2.4	26,515	2.4	28,524	2.6	30,879	2.8	160
2021	January	32,324	3.0	33,823	3.1	35,185	3.2	36,633	3.3	38,619	3.5	213
2021	February	29,038	2.7	29,973	2.8	31,199	2.9	33,392	3.0	35,052	3.2	199
2021	March	27,303	2.5	28,192	2.6	29,527	2.7	31,603	2.8	33,213	3.0	161
2021	April	37,626	3.5	39,725	3.7	42,341	3.9	45,943	4.1	48,196	4.4	285
2021	May	34,776	3.2	36,319	3.4	38,457	3.5	42,261	3.8	45,447	4.2	243
2021	June	34,107	3.2	35,155	3.2	37,966	3.5	41,032	3.7	43,346	4.0	344
2021	July	36,861	3.4	39,257	3.6	42,169	3.9	45,978	4.1	47,931	4.4	304
2021	August	38,933	3.6	40,859	3.8	42,760	3.9	47,284	4.2	50,977	4.7	359
2021	September	35,345	3.3	36,795	3.4	39,436	3.6	43,067	3.9	45,309	4.1	294
2021	October	38,826	3.6	41,330	3.8	44,234	4.1	49,031	4.4	51,378	4.7	375
2021	November	41,590	3.8	44,729	4.1	47,898	4.4	52,006	4.7	55,725	5.1	334
2021	December	31,673	2.9	33,795	3.1	36,775	3.4	41,439	3.7	44,696	4.1	315
2022	January	37,599	3.5	40,538	3.7	43,240	4.0	47,641	4.3	49,760	4.6	397
2022	February	37,354	3.4	40,608	3.7	42,909	3.9	47,770	4.3	50,366	4.6	440
2022	March	30,876	2.9	32,831	3.0	35,040	3.2	38,930	3.5	40,592	3.7	252
2022	April	54,932	5.1	58,385	5.4	61,737	5.7	69,076	6.2	72,682	6.6	386
2022	May	52,951	4.9	56,397	5.2	59,386	5.4	65,199	5.8	68,726	6.3	445

Children dental contacts by SIMD quintile: Number of children and % of child population seen by GDS/PDS; by payment month and

SIMD quintile; Scotland

		SIMD 1 - most deprived		SIMD 2		SIMD 3		SIMD 4		SIMD 5 - least deprived		SIMD Unknown
Year	Month	No. of patients	% of pop.	No. of patients	% of pop.	No. of patients	% of pop.	No. of patients	% of pop.	No. of patients	% of pop.	No. of patients
2019	January	20,842	9.1	20,048	9.9	20,422	10.7	23,013	11.3	24,431	12.1	200
2019	February	19,154	8.3	18,855	9.3	19,173	10.1	21,901	10.7	23,248	11.5	165
2019	March	20,845	9.1	20,100	9.9	19,980	10.5	22,802	11.2	23,914	11.9	236
2019	April	17,596	7.7	17,812	8.8	17,933	9.4	20,934	10.3	21,603	10.7	201
2019	May	20,024	8.7	19,569	9.6	20,211	10.6	22,973	11.3	24,630	12.2	197
2019	June	17,332	7.5	16,794	8.3	17,748	9.3	19,962	9.8	21,377	10.6	179
2019	July	17,297	7.5	17,587	8.6	17,944	9.4	20,643	10.1	21,265	10.5	194
2019	August	19,014	8.3	18,778	9.2	19,794	10.4	22,846	11.2	24,552	12.2	197
2019	September	18,052	7.9	17,680	8.7	17,586	9.2	20,022	9.8	21,173	10.5	168
2019	October	20,724	9.0	20,169	9.9	20,318	10.7	23,729	11.6	24,773	12.3	248
2019	November	22,323	9.7	21,085	10.4	20,690	10.9	23,549	11.5	24,173	12.0	208
2019	December	12,386	5.4	11,847	5.8	12,842	6.7	15,198	7.4	16,784	8.3	167
2020	January	20,660	9.0	19,836	9.8	19,764	10.4	22,880	11.1	23,971	11.9	171
2020	February	18,529	8.1	17,992	8.9	17,870	9.4	21,228	10.3	22,406	11.2	131
2020	March	8,726	3.8	8,494	4.2	8,553	4.5	9,637	4.7	10,641	5.3	99
2020	April	746	0.3	683	0.3	803	0.4	876	0.4	422	0.2	11
2020	May	319	0.1	304	0.1	267	0.1	331	0.2	215	0.1	1
2020	June	839	0.4	639	0.3	503	0.3	538	0.3	575	0.3	3
2020	July	4,428	1.9	3,503	1.7	2,947	1.6	3,428	1.7	3,636	1.8	40
2020	August	4,165	1.8	3,357	1.7	3,278	1.7	3,851	1.9	4,681	2.3	42
2020	September	4,702	2.1	4,138	2.0	4,165	2.2	4,615	2.2	5,484	2.7	54
2020	October	5,397	2.4	4,932	2.4	5,389	2.8	6,387	3.1	7,345	3.7	63
2020	November	5,722	2.5	5,629	2.8	5,826	3.1	7,002	3.4	7,888	3.9	41
2020	December	4,510	2.0	4,177	2.1	4,296	2.3	5,236	2.5	6,282	3.1	39
2021	January	6,126	2.7	6,063	3.0	6,451	3.4	7,359	3.6	8,538	4.3	57
2021	February	5,636	2.5	5,569	2.7	5,840	3.1	7,093	3.5	7,973	4.0	55

2021	March	5,541	2.4	5,044	2.5	5,500	2.9	6,535	3.2	7,261	3.6	32
2021	April	7,579	3.3	7,804	3.9	8,648	4.6	10,338	5.0	11,542	5.8	102
2021	May	7,319	3.2	7,234	3.6	7,592	4.0	9,228	4.5	10,496	5.2	60
2021	June	6,762	3.0	6,804	3.4	7,385	3.9	8,889	4.3	9,657	4.8	86
2021	July	7,754	3.4	7,877	3.9	8,804	4.6	10,604	5.2	11,682	5.8	80
2021	August	8,502	3.7	8,477	4.2	9,242	4.9	11,152	5.4	12,957	6.5	117
2021	September	7,359	3.2	7,168	3.5	7,595	4.0	9,247	4.5	10,280	5.1	75
2021	October	8,462	3.7	8,568	4.2	9,454	5.0	11,641	5.7	12,653	6.3	105
2021	November	9,473	4.1	9,701	4.8	10,117	5.3	11,989	5.8	13,802	6.9	112
2021	December	6,323	2.8	6,447	3.2	7,149	3.8	8,974	4.4	10,301	5.1	82
2022	January	8,155	3.6	8,283	4.1	8,882	4.7	10,689	5.2	11,289	5.6	100
2022	February	8,487	3.7	8,702	4.3	9,167	4.8	11,276	5.5	12,342	6.2	123
2022	March	7,592	3.3	7,576	3.7	7,994	4.2	9,698	4.7	10,546	5.3	61
2022	April	13,541	5.9	13,169	6.5	13,994	7.4	17,575	8.6	18,607	9.3	96
2022	May	13,088	5.7	12,618	6.2	13,436	7.1	16,121	7.9	17,106	8.5	106

Adults' dental contacts by SIMD quintile: Number of adults and % of adult population seen by GDS/PDS; by payment month and SIMD quintile; Scotland

		SIMD 1 - most deprived		SIMD 2		SIMD 3		SIMD 4		SIMD 5 - least deprived		SIMD Unknown
Year	Month	No. of patients	% of pop.	No. of patients	% of pop.	No. of patients	% of pop.	No. of patients	% of pop.	No. of patients	% of pop.	No. of patients
2019	January	67,651	7.9	75,686	8.6	80,510	8.9	85,208	9.4	86,795	9.7	599
2019	February	57,854	6.8	64,831	7.3	67,924	7.5	72,629	8.0	73,134	8.2	436
2019	March	63,251	7.4	69,708	7.9	73,254	8.1	77,634	8.6	78,387	8.8	537
2019	April	55,716	6.5	62,330	7.1	65,395	7.3	70,498	7.8	70,318	7.9	507
2019	May	65,865	7.7	73,533	8.3	78,138	8.7	82,935	9.2	85,124	9.6	540
2019	June	58,244	6.8	64,423	7.3	67,652	7.5	72,233	8.0	72,211	8.1	503
2019	July	56,718	6.6	62,831	7.1	66,251	7.3	70,605	7.8	70,962	8.0	484
2019	August	62,825	7.3	69,139	7.8	73,915	8.2	78,472	8.7	79,991	9.0	508
2019	September	57,764	6.7	64,656	7.3	68,354	7.6	72,958	8.1	74,121	8.3	554
2019	October	64,036	7.5	71,183	8.1	75,156	8.3	79,771	8.8	80,451	9.0	531

2019	November	69,350	8.1	76,821	8.7	80,506	8.9	84,264	9.3	84,023	9.4	651
2019	December	43,434	5.1	48,109	5.4	51,181	5.7	55,429	6.1	56,985	6.4	427
2020	January	67,651	7.9	76,674	8.7	81,491	9.0	86,765	9.5	87,196	9.8	627
2020	February	56,929	6.7	63,693	7.2	67,552	7.5	72,521	8.0	72,859	8.2	442
2020	March	32,922	3.9	35,277	4.0	37,641	4.2	39,679	4.4	39,036	4.4	281
2020	April	2,265	0.3	2,189	0.2	2,141	0.2	2,138	0.2	1,444	0.2	28
2020	May	1,973	0.2	1,697	0.2	1,386	0.2	1,464	0.2	1,075	0.1	12
2020	June	4,808	0.6	4,416	0.5	4,054	0.4	4,049	0.4	3,917	0.4	16
2020	July	25,524	3.0	24,450	2.8	22,881	2.5	22,955	2.5	20,975	2.3	141
2020	August	22,284	2.6	21,989	2.5	21,276	2.4	21,980	2.4	21,721	2.4	173
2020	September	23,353	2.7	23,569	2.7	23,785	2.6	25,170	2.8	25,177	2.8	198
2020	October	27,906	3.3	27,997	3.2	28,103	3.1	29,160	3.2	29,858	3.3	181
2020	November	25,281	3.0	25,685	2.9	26,656	3.0	28,368	3.1	29,222	3.3	160
2020	December	21,293	2.5	21,885	2.5	22,219	2.5	23,288	2.6	24,597	2.8	121
2021	January	26,198	3.1	27,760	3.2	28,734	3.2	29,274	3.2	30,081	3.4	156
2021	February	23,402	2.7	24,404	2.8	25,359	2.8	26,299	2.9	27,079	3.0	144
2021	March	21,762	2.5	23,148	2.6	24,027	2.7	25,068	2.8	25,952	2.9	129
2021	April	30,047	3.5	31,921	3.6	33,693	3.7	35,605	3.9	36,654	4.1	183
2021	May	27,457	3.2	29,085	3.3	30,865	3.4	33,033	3.6	34,951	3.9	183
2021	June	27,345	3.2	28,351	3.2	30,581	3.4	32,143	3.5	33,689	3.8	258
2021	July	29,107	3.4	31,380	3.6	33,365	3.7	35,374	3.9	36,249	4.1	224
2021	August	30,431	3.6	32,382	3.7	33,518	3.7	36,132	4.0	38,020	4.3	242
2021	September	27,986	3.3	29,627	3.4	31,841	3.5	33,820	3.7	35,029	3.9	219
2021	October	30,364	3.6	32,762	3.7	34,780	3.9	37,390	4.1	38,725	4.3	270
2021	November	32,117	3.8	35,028	4.0	37,781	4.2	40,017	4.4	41,923	4.7	222
2021	December	25,350	3.0	27,348	3.1	29,626	3.3	32,465	3.6	34,395	3.9	233
2022	January	29,444	3.4	32,255	3.7	34,358	3.8	36,952	4.1	38,471	4.3	297
2022	February	28,867	3.4	31,906	3.6	33,742	3.7	36,494	4.0	38,024	4.3	317
2022	March	23,284	2.7	25,255	2.9	27,046	3.0	29,232	3.2	30,046	3.4	191
2022	April	41,391	4.8	45,216	5.1	47,743	5.3	51,501	5.7	54,075	6.1	290
2022	May	39,863	4.7	43,779	5.0	45,950	5.1	49,078	5.4	51,620	5.8	339
